# Unusual genome complexity in *Lactobacillus salivarius* JCM1046

**DOI:** 10.1186/1471-2164-15-771

**Published:** 2014-09-08

**Authors:** Emma J Raftis, Brian M Forde, Marcus J Claesson, Paul W O’Toole

**Affiliations:** School of Microbiology University College Cork, Cork, Ireland; Alimentary Pharmabiotic Centre, University College Cork, Cork, Ireland

**Keywords:** *Lactobacillus salivarius*, Megaplasmid, Multireplicon, Linear plasmid, Tn6224, Conjugative transposon

## Abstract

**Background:**

*Lactobacillus salivarius* strains are increasingly being exploited for their probiotic properties in humans and animals. Dissemination of antibiotic resistance genes among species with food or probiotic-association is undesirable and is often mediated by plasmids or integrative and conjugative elements. *L. salivarius* strains typically have multireplicon genomes including circular megaplasmids that encode strain-specific traits for intestinal survival and probiotic activity. Linear plasmids are less common in lactobacilli and show a very limited distribution in *L. salivarius*. Here we present experimental evidence that supports an unusually complex multireplicon genome structure in the porcine isolate *L. salivarius* JCM1046.

**Results:**

JCM1046 harbours a 1.83 Mb chromosome, and four plasmids which constitute 20% of the genome. In addition to the known 219 kb *repA*-type megaplasmid pMP1046A, we identified and experimentally validated the topology of three additional replicons, the circular pMP1046B (129 kb), a linear plasmid pLMP1046 (101 kb) and pCTN1046 (33 kb) harbouring a conjugative transposon. pMP1046B harbours both plasmid-associated replication genes and paralogues of chromosomally encoded housekeeping and information-processing related genes, thus qualifying it as a putative chromid. pLMP1046 shares limited sequence homology or gene synteny with other *L. salivarius* plasmids, and its putative replication-associated protein is homologous to the RepA/E proteins found in the large circular megaplasmids of *L. salivarius.* Plasmid pCTN1046 harbours a single copy of an integrated conjugative transposon (Tn6224) which appears to be functionally intact and includes the tetracycline resistance gene *tetM*.

**Conclusion:**

Experimental validation of sequence assemblies and plasmid topology resolved the complex genome architecture of *L. salivarius* JCM1046. A high-coverage draft genome sequence would not have elucidated the genome complexity in this strain. Given the expanding use of *L. salivarius* as a probiotic, it is important to determine the genotypic and phenotypic organization of *L. salivarius* strains. The identification of Tn6224-like elements in this species has implications for strain selection for probiotic applications.

**Electronic supplementary material:**

The online version of this article (doi:10.1186/1471-2164-15-771) contains supplementary material, which is available to authorized users.

## Background

*Lactobacillus salivarius* [1] is a member of the indigenous microbiota of the oral cavity and the gastrointestinal tract (GIT) of both humans and animals [2, 3], and has also been isolated from human breast milk [4]. The probiotic and immunomodulatory activity of *L. salivarius* strains has been recently reviewed [5] and are considered to be strain-specific traits [6]. Strains of *L. salivarius* are genetically diverse [7] and harbour distinctive multireplicon genomes. The first genome of this species to be published [8, 9] was that of the well-characterised strain *L. salivarius* UCC118 [1, 10–13] whose megaplasmid pMP118 (242 kb) encodes genes involved in GI tract survival, fitness and probiotic activity [9–11]. *L. salivarius* strains from a range of environmental sources harbour diverse circular megaplasmids [7, 12]. At least 10 additional *L. salivarius* genomes have been sequenced since that of strain UCC118; three of these have been completed (strains CECT 5713 [14] NIAS840 [15] and SMXD51 [16]) with two being finished to a draft quality status [17, 18].

Unlike circular plasmids, linear plasmids are rarely observed in lactobacilli [12] but often confer advantageous phenotypes to their hosts [19, 20] and have been extensively studied in *Streptomyces* [21, 22], *Borrelia* [23] and *Bacillus* [24]. Linear phage genomes are also harboured by strains of *Escherichia coli* [25], *Yersinia enterocolitica* [26], *Klebsiella oxytoca* [27] as well as the probiotic cheese strain *Lactobacillus paracasei* NFBC 338 [28]. Prior to the discovery of linear megaplasmids in *L. salivarius* [12], a 150 kb linear plasmid was identified in *Lactobacillus gasseri* CNRZ222 [29]; but no characterization of the plasmid was performed. We previously identified linear megaplasmids in two porcine *L. salivarius* isolates, JCM1046 and JCM1047, and one human intestinal isolate AH43348 [12].

The conjugative transposon (CTs) Tn916 (18.5 kb) [30] and other Tn916-like elements are highly promiscuous [31], both in the lab and in natural environments [32]. They have demonstrated intra- and interspecies transfer from *Lactococcus lactis* [33] and *Lactobacillus paracasei* [34] food strains; and between streptococcal species in dental biofilms [35]. There is a growing concern that commensal bacteria may act as natural reservoirs for antibiotic resistance determinants [36] and may be responsible for transfer of antibiotic resistance to pathogens and opportunistic pathogens [37]. In addition to the introduction of additional functional modules to the host cell, CTs have further potential to influence natural selection within a bacterial population [38]. There is therefore a growing need to characterize these mobile elements, particularly in species used in food or as probiotics.

Here we present experimental evidence for a highly unusual genome architecture in *L. salivarius* JCM1046, a strain that harbours multiple extrachromosomal replicons of varying sizes and topologies and which has an enhanced ability to withstand the stresses associated with GIT survival [11]. The present study describes an unprecedented level of genome complexity in *L. salivarius*.

## Results and discussion

### Discovery of circular and linear extrachromosomal elements in *L. salivarius*JCM1046

**Figure 1:**
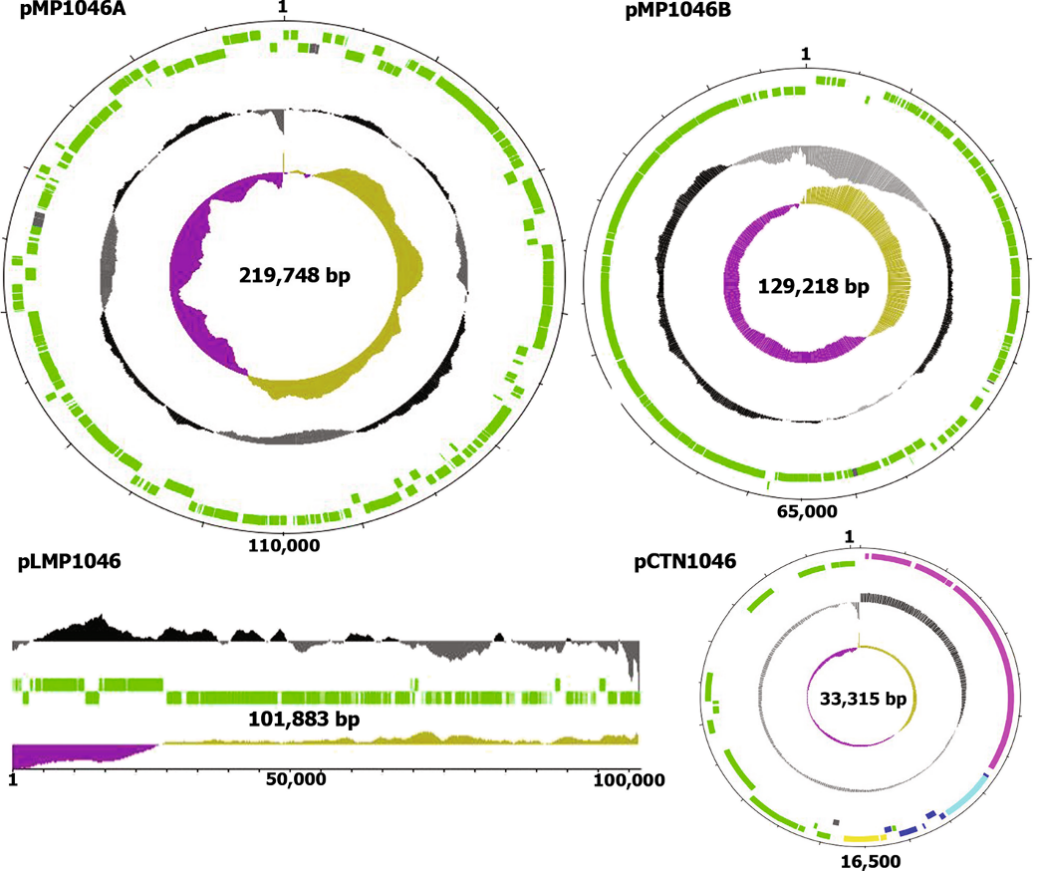
**Genome atlas of the plasmids of** ***L. salivarius*** **JCM1046.** A graphical representation of each plasmid in the *L. salivarius* JCM1046 genome was generated using DNAPLOTTER [39]. Genes on the forward and reverse strands (green); pseudogenes (grey blocks); GC% (black above mean and grey below mean); GC skew (mustard above mean and purple below mean) are illustrated for each replicon. Genes encoded by the plasmid backbone of pCTN1046 are also green, the genes present on the integrated conjugative transposon Tn6224 are represented as follows: conjugative transfer (pink), accessory genes (turquoise), transcriptional regulation (dark blue) and recombination (yellow).

**Figure 2:**
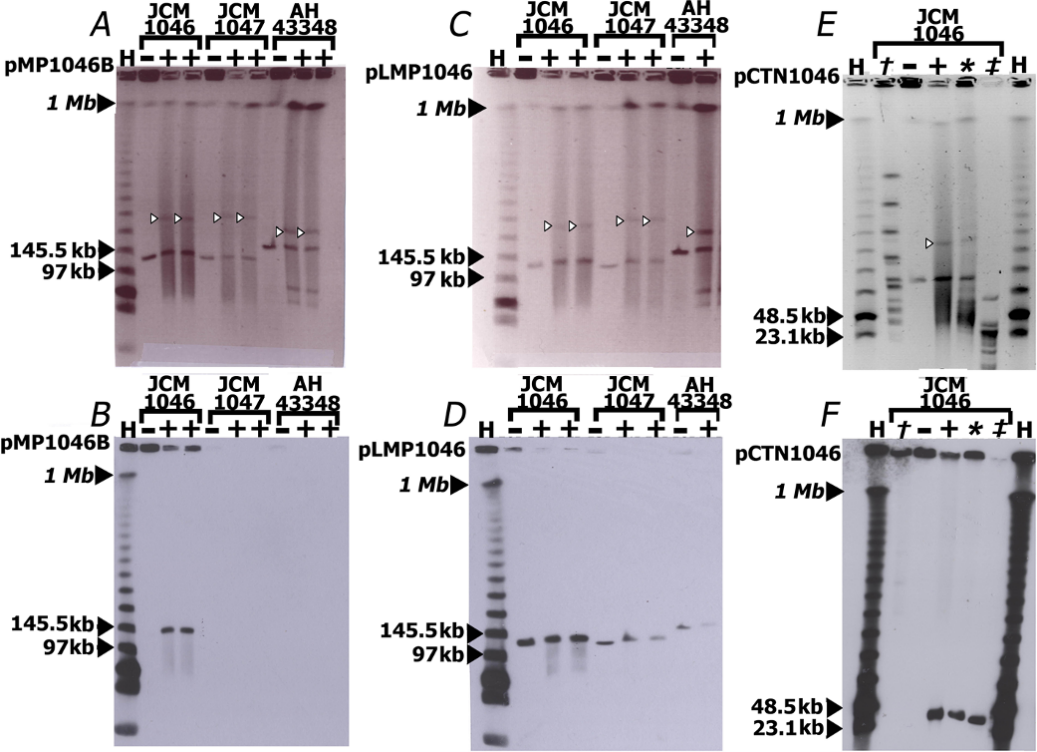
**Confirmation of the genome architecture of** ***L. salivarius*** **JCM1046. (A, C and** **D)** PFGE gels of enzyme-treated gDNA of strains JCM1046, JCM1047 and AH43348. Corresponding Southern Hybridizations using replicon-specific probes are shown directly below each gel **(B, D,** **and F)**. The probes used for the Southern Hybridizations targeted the following genes: the *repB* gene of pMP1046B **(B)**, an endonuclease gene in pLMP1046 **(D)** and a region spanning the *int*-*xis* genes of pCTN1046 **(F)**. None of the probes employed showed cross hybridisation with non-target replicons. S1 nuclease (+), SmaI (†), SphI (_*_), PstI (‡) were used individually or in combination to determine the plasmid profiles of each strain. Untreated samples of gDNA are denoted by (−). Closed-black arrowheads indicate λ DNA concatamers used as size standards **(H) (A-F)**. Chromosomal DNA bands of each strain are seen migrating to the equivalent of the 1 Mb marker **(A, C and E)**. Open-black arrows indicate the S1 nuclease-linearised *repA* megaplasmids in each strain examined **(A, C and E)**. A *repB*-type megaplasmid was found to be present in strain JCM1046 but absent from strains JCM1047 and AH43348 **(A and** **B)**. Both S1-treated and untreated gDNA samples of JCM1046, JCM1047 and AH43348 show the presence of linear plasmids of 140 kb, 140 kb and 175 kb respectively **(C)**, each of which hybridise to a pLMP1046-derived probe **(D)**. S1-nuclease, SphI and PstI were independently used to linearise pCTN1046 (33 kb) **(E)**. A probe based on the *int* and *xis* genes of pCTN1046 binds to the linear form of pCTN1046 **(F)**. pCTN1046 does not have a SmaI site and is retained in the well in its circular form in the SmaI-digested sample.

Sequencing revealed that *L. salivarius* JCM1046 contains five replicons (Table 1): a 1.836 Mb chromosome, two circular megaplasmids of 219 and 129 kb, a linear megaplasmid of 101 kb, and a 33 kb plasmid harbouring an integrated conjugative transposon (Figure 1). The complexity of this genome configuration presented extraordinary challenges for genome assembly, described below. Experimental validation of the genome structure is presented in Figure 2. *L. salivariu*s strains JCM1047 and AH43348 were known to harbour linear megaplasmids that were presumed to be related to pLMP1046 [12] and were therefore included in these experiments.

**Table 1:** **General genome features of** ***L. salivarius*** **JCM1046**

Feature	Chromosome	pMP1046A	pMP1046B	pLMP1046	pCTN1046
Replicon size (bp)	1,836,297	219,748	129,218	101,883	33,315
GC Content (%)	33.1	32.04	33.87	30.91	34.89
Topology	Circular	Circular	Circular	Linear	Circular
% of genome size	79.1	9.4	5.5	4.3	1.4
Coding genes	1705	214	159	112	40
Coding density (%)	83.3%	80.7%	83.6%	82.6%	76%
rRNA operons	7	0	0	0	0
tRNAs	75	0	2	0	0
Pseudogenes	60	15	2	0	1

Our original study that identified pMP1046A (then designated pMP1046 [12]) in strain JCM1046 estimated its size as 230 kb, based on Pulsed Field Gel Electrophoresis (PFGE) [12]. However, the assembled sequence data revealed pMP1046A as closer to 220 kb in size. A combination of restriction digestion, PFGE and Southern hybridisation was used to validate the size of pMP1046A. *ApaI* was used to linearise the replicon prior to PFGE and Southern Blot analysis. Probes associated with the replication origin of pMP1046A hybridised to a band that migrated to a constant position between the 194 kb and 242.5 kb linear λ DNA markers, which was in keeping with the expected 219,748 bp size indicated by DNA sequencing.

We identified two novel plasmids pMP1046B and pCTN1046 from the genome sequence. A large contig (~130 kb) was assembled that could not be experimentally determined to form part of either the chromosome or previously described plasmid content of strain JCM1046 [12]. This contig harboured plasmid-associated replication and maintenance proteins. A PCR product off the ends of this contig was generated and subsequently sequenced (data not shown) which proved that the assembled contig was circular in the cell, and it was designated pMP1046B. Under the PFGE conditions that are routinely used to visualise the plasmid content of *L. salivarius* strains, pM1046B had previously gone undetected [9, 40] possibly because it was masked by the linear replicon pLMP1046 [12].

We employed restriction digestion and S1 nuclease treatment in conjunction with PFGE and Southern Blot analysis to confirm the sizes and topologies of the plasmids present in JCM1046. Figure 2 panels A and B illustrate the identification of a *repB*-type megaplasmid in JCM1046, panels C and D display the linear plasmids of JCM1046, JCM1047 and AH43348, and panels E and F illustrate the size and topolgy of pCTN1046. Chromosomal DNA bands of strains JCM1046, JCM1047 and AH43328 migrate to the equivalent of the 1 Mb marker (Figure 2 panels A, C and E). S1 Nuclease preferentially nicks and linearises megaplasmids due to inherent torsional stresses [41]. The linearised form of the *repA*-type circular megaplasmids of the *L. salivarius* strains are indicated by the open black arrows in Figure 2 panels A, C and E.

When an increased band intensity or band width is observed in a PFGE gel, it is often indicative of the presence of linear DNA, high copy number extrachromosomal elements or co-migrating bands of similarly sized DNA fragments [42]. Strain JCM1046 gDNA revealed high-intensity bands in the S1-treated sample at a position just below the 145.5 kb lambda DNA marker. This band represents the overlapping linear forms of pMP1046B and pLMP1046. In the untreated sample of JCM1046, the circular form of pMP1046B is retained in the well; therefore the *repB* gene probe binds only to the well but not to the migrating linear plasmid pLMP1046 (Figure 2 panel B). However, in the S1-nuclease treated gDNA sample of JCM1046, the *repB* probe hybridised strongly to the overlapping pLMP1046/pMP1046B bands (Figure 2 panel B), thereby confirming that the discrete replicons pLMP1046 and pMP1046B appear as one overlapping 120 kb band in their linear forms (Figure 2 panel B). The *repB* probe did not hybridise to the lanes containing JCM1047 or AH43348 gDNA, indicating that these strains lack a second *repB*-type circular megaplasmid (Figure 2 panel B). The presence of a second circular megaplasmid has also been reported in strains NIAS840 and SMXD51, both of these strains being of animal origin [15, 16].

Both S1-treated and untreated gDNA samples of JCM1046, JCM1047 and AH43348 show the presence of linear plasmids: pLMP1046 (140 kb), pLMP1047 (140 kb) and pLMP43348 (175 kb) respectively (Figure 2, panels A and C). Each of the linear plasmids hybridised to a gene probe derived from the pLMP1046 sequence (Figure 2D).

### A conjugative transposon in *L. salivarius*JCM1046

We further identified a 33 kb plasmid in strain JCM1046 that was not previously observed in the plasmid profile of strain JCM1046 [12, 40] and that was identified here by *de novo* scaffold assembly and designated pCTN1046. It harbours a Tn916-like element and was experimentally determined to have a circular topology. *In silico* analysis was first used to identify restriction enzymes whose use would resolve the chromosomal DNA of JCM1046 from that of pCTN1046. *SphI* and *PstI* each cut the chromosome multiple times, while linearising pCTN1046. Following treatment, pCTN1046 is visible as a band which migrates to a position between the 23.1 kb and 48.5 kb, in keeping with the assembled 33 kb size of pCTN1046 (Figure 2E). The chromosome of JCM1046 has multiple *SmaI* restriction sites, while pCTN1046 has none. The multiple DNA bands in the *SmaI*-treated gDNA sample (Figure 2E) are chromosomal fragments, while the uncut circular form of pCTN1046 was retained in the well. A probe spanning the *int* and *xis* genes of pCTN1046 hybridised strongly to the 33 kb bands in the S1-nuclease, *SphI* and *PstI* treated samples of JCM1046 (Figure 2 F). Similarly, the same probe hybridised to the circular form of pCTN1046 retained in the well of the *SmaI*-treated sample, but did not hybridise to the migrating chromosomal bands (Figure 2F). The same pattern of hybridisation was obtained when the experiment was repeated with a probe based on the *tetM* gene harboured by pCTN1046 (data not shown). Although Tn916-like elements have been shown to insert at a single site in some species, in almost all bacterial hosts they insert at multiple sites [43]. Our data indicate that the conjugative transposon in strain JCM1046 is integrated at a single site in pCTN1046 and is absent from the rest of the genome.

### General genome features of *L. salivarius*JCM1046

The unusual genome complexity of JCM1046 raised questions about gene distribution by replicon. Bioinformatic analysis identified 1,705 coding sequences in the chromosome, a coding density of 83.3% (Table 1). Biological functions could not be assigned to 360 of these protein coding sequences. The chromosome of *L. salivarius* JCM1046 contains 60 pseudogenes (Additional file 1). Seven rRNA operons were identified on the chromosome, as well as 76 tRNA genes for all 20 amino acids. The chromosome has an average GC content of 33.1%, with three regions displaying atypical GC content relative to the rest of the genome (see below).

The largest of the plasmids pMP1046A has a coding density of 80.7%. 214 coding sequences were identified, 79 of which were for hypothetical proteins. pMP1046A contains 15 pseudogenes (Additional file 1). The gene content of pMP1046A will be discussed in detail below.

We identified 159 coding regions in pMP1046B, though biological function could only be assigned to 29.7%, the vast majority (110/158) of genes remaining cryptic. The GC% content of pMP1046B (33.87%) correlates well with the 33.1% GC content of the JCM1046 chromosome (Table 1) suggesting long-term adaptation to the host cell, or acquisition from a bacterium with a similar % GC content. In addition to harbouring plasmid-associated replication machinery, pMP1046B harbours additional housekeeping and information-related genes, thus fulfilling the criteria for extrachromosomal elements known as chromids [44]. pMP1046B encodes two tRNA genes, tRNA (Gln) (LSJ_3064) and tRNA (Ser) (LSJ_3066) but these genes are not uniquely present on pMP1046B i.e. they are paralogs of chromosomally encoded genes. Gene duplication can offer a level of genomic redundancy to a strain that is adapting to a new environment [45], and the tRNA genes encoded by pMP1046B may enable JCM1046 to respond more rapidly to changing environmental conditions. pLMP1046 harbours 112 coding sequences, none of which were pseudogenes. However, 85 of the predicted coding sequence products were annotated as hypothetical proteins, some of which may represent remnants of functional genes. The average GC content of pLMP1046 (30.9%) is significantly lower than that of the JCM1046 chromosome (33.1%), implying these replicons experienced distinct evolutionary histories and that pLMP1046 may be a recent acquisition.

PFGE analysis predicted the size of pLMP1046 to be approximately 130 kb (this study), but sequencing revealed a replicon that was 102 kb. It is reasonable to assume that this discrepancy and the lack of identifiable terminal inverted repeats (TIR) (discussed below) is an assembly artifact due to omission of the presumptive repeat sequences in the terminal regions of pLMP1046. The problems faced in the sequencing of the telomeres of linear elements are well recognised [46].

In keeping with the guidelines outlined by Roberts *et al.* [47] the novel conjugative transposon contained within pCTN1046 was designated Tn6224. *In silico* analysis predicted a coding density of 76% for pCTN1046. Thirty-nine coding sequences were identified (Table 1), the majority of which (21/39) are linked to the integrated transposon. The sole pseudogene harboured by this replicon lies outside the Tn6224 region and shows similarity to nitroreductase family proteins. The plasmid backbone of pCTN1046 has an average GC content of 30.8%, whereas Tn6224 has an average GC content of 38.6%. Unsurprisingly, this suggests that Tn6224 was most likely acquired *via* horizontal gene transfer (HGT). Insertion of Tn916-like elements is not random, with the insertion sites differing from species to species [38], but generally displaying a distinct preference for target sites which are A-T rich and that have a limited homology with the ends of the element [43]. As only one copy of Tn6224 was found in the genome of JCM1046, a putative consensus of the target sequence in *L. salivarius* could not be determined. Accounting for the potential presence of coupling sequences, the 35 bp that flanked either end of Tn6224 was examined to determine if the target sites in *L. salivarius* are in keeping with those generally described for these elements [38]. The AT content of the sequences upstream and downstream of Tn6224 were found to be 97.1% and 85.7% respectively, indicating that the target site for Tn6224 is likely to be similar to those of other species [38].

### Phage, transposases and CRISPR regions

PHAST [48] identified two regions of bacteriophage-related DNA in the genome of JCM1046, both found on the chromosome of JCM1046. In addition to a 22.6 kb remnant prophage that spans residues 1378015–1400296 bp, an intact 28,541 kb prophage was also identified on the chromosome which spans residues 1439831–1444300 bp. At 43.7%, the remnant prophage is one of the three regions of atypical GC content.

102 transposases (including 22 pseudogenes), representing eight IS families were found distributed across four of the five replicons of strain JCM1046. The distribution of transposases is detailed in Additional file 2.

Clusters of regularly interspersed short palindromic repeats (CRISPRs) and CRISPR-associated genes (*cas* genes) provide the host with acquired and heritable resistance against genetic transformation, phage and plasmid proliferation [49]. One CRISPR associated system (cas) was identified on the chromosome of JCM1046 at position 810173–812140 bp, consisting of a 1059 bp repeat locus composed of a 36 bp direct repeat and 26 spacers. This CRISPR region is immediately upstream of the gene encoding Cas2 and immediately downstream of eight additional CRISPR-associated protein coding genes.

### Replication of extrachromosomal elements

**Figure 3:**
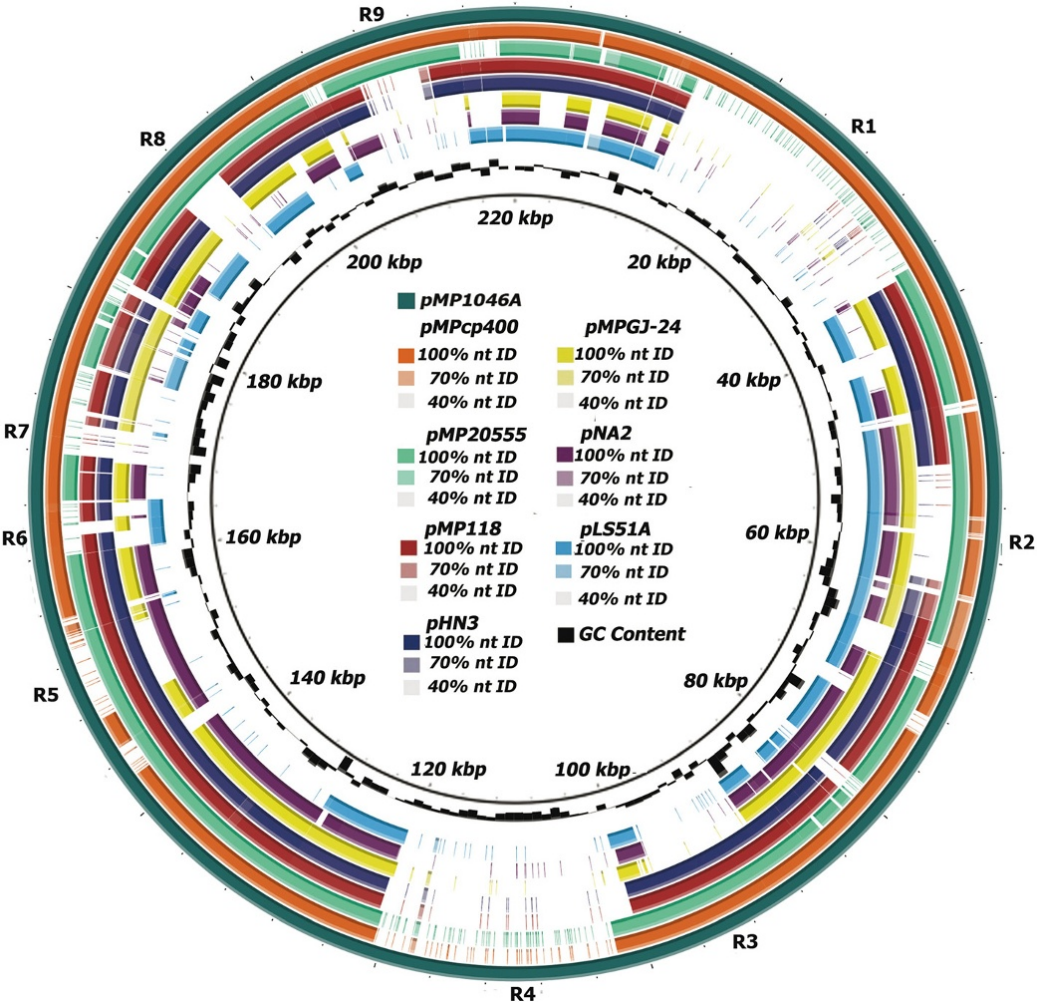
**A comparison eight** ***repA*** **-type megaplasmids of** ***L. salivarius.*** A BLAST atlas diagram of eight *repA*-type megaplasmids of *L. salivarius* was generated using BLAST Ring Image Generator **(**BRIG) [51], using pMP1046A as the reference replicon (the outer dark green ring). Working inwards from pMP1046A, the next seven rings represent query *repA*-type plasmids of *L. salivarius* strains: cp400, pMP20555, pMP118, pHN3, pMPGJ-24, pNA2, pLS51A. When the completed or circularised version of the *repA*-type megaplasmid was not available (*L. salivarius* cp400 [18] and *L. salivarius* DSM20555), all available sequence data for each strain was mapped to pMP1046A. Regions of diversity between the *repA*-type megaplasmids are indicated by the labels R1-R9. The GC% of pMP1046A was projected onto the mapped plasmid sequences (black ring) and sits outside the molecular clock surrounding the figure legend at the centre of the figure.

The replication region of pMP1046A extends from LSJ_2000 to LSJ_2006 (6449 bp). The gene content and organisation of the replication region of pMP1046A is highly similar to (98% nt identity (ID)) that of pMP118 [9] and to those of other sequenced *L. salivarius* strains (Figure 3). pMP1046A is likely to replicate by theta-form replication [50].

The predicted replication region of pMP1046B spans residues 128175–1974 bp of the plasmid. This region includes a *repA* gene (LSJ_3160) at the position of a switch in GC skew that is characteristic of replication origins [52]. LSJ_3160 shares 36-56% aa ID with *L. salivarius* RepA protein sequences. The RepA protein of pMP1046B also displays 40% aa ID to the RepA protein of the pig isolate *Lactobacillus reuteri* ATCC 53608 [53]. The second gene in the pMP1046B *ori* region, LSJ_3000 encodes a predicted partitioning/copy control protein, RepB.

Analysis of pLMP1046 indicates that it shares limited sequence homology or gene synteny with linear replicons of other species. However, given the lack of sequenced counterparts in other lactobacilli, the absence of homologous genes in databases is unsurprising. Replication is commonly initiated from one or more internal *ori* sites in linear plasmids and proceeds bidirectionally towards the telomeres [54–56]. Our previous study indicated that the linear plasmids of *L. salivarius* did not harbour the *repA* and *repE* genes encoded by the circular *repA*-type megaplasmids of *L. salivarius* [12], and thus it was presumed that pLMP1046 utilised an alternate mode of replication to the circular plasmids of *L. salivarius* [12]. Sequence analysis identified two plasmid-associated replication genes encoded by pLMP1046, LSJ_4017 (nt 25084–26103) and LSJ_4096 (nt 89781–91007). LSJ_4017 exhibits 39-41% aa ID with proteins annotated as either RepE or RepA in the circular megaplasmids of *L. salivarius*. This level of sequence homology was not high enough to cause cross hybridisation between the replication genes of pMP118 and the *repA/E* gene identified in pLMP1046, thus accounting for the observations of our previous study [12]. LSJ_4096 encodes a putative RepB-like replication initiator protein. The replication origins of *Streptomyces* linear plasmids are comprised of helicase-like *rep* genes and interons [22], while the replication *ori* of N15 is located within the r*epA* gene, which acts as a multifunctional protein combining primase, helicase and origin-binding activities [57]. RepA boxes were not identified in the proximity of either the *repA* or *repB* genes of pLMP1046; however, the genomic region immediately upstream of the *repA* coincides with a switch in GC skew. This suggests that the *repA* gene lies within the putative *ori* region of pLMP1046.

The mechanism that pLMP1046 uses to prevent the progressive shortening of their telomeres after each cycle of replication is unknown. It is possible it employs a circular mode, as in some *Streptomyces* linear plasmids [58], but it is more plausible that the sequence of pLMP1046 is missing sections of its terminal regions due to a sequencing or assembly artefact. Further analysis of the terminal regions of pLMP1046 will be required to fully elucidate the mechanism involved in the replication of *L. salivarius* linear plasmids.

There are two replication associated genes harboured by the plasmid backbone of pCTN1046 which are separated by approximately 6 kb. LSJ_5030c shares 52% aa ID with a replication-associated protein in *Lactobacillus amylovorus* GRL 1112. LSJ_5035c encodes the plasmid associated replication protein, RepB, the gene for which coincides with the position of a switch in GC skew, and is therefore the presumed to be the replication origin of pCTN1046. LSJ_5035c shares 36% aa ID with the RepB protein of *L. lactis* subsp. *cremoris* TIFN1 and 100% aa ID to a replication initiation protein in the 30.6 kb plasmid pLS51C in *L. salivarius* SMXD51.

#### Plasmid maintenance

Several of the JCM1046 plasmids encode genes implicated in plasmid incompatibility. Three of the plasmids (pMP1046B, pLMP1046 and pCTN1046) encode a *repB*-like gene, two (pMP1046A and pMP1046B) encode *repE*-like genes and two (pMP1046A and pLMP1046) encode *repA*-like genes. However the presumptive replication regions of the co-resident plasmids display low levels of sequence ID with the highest nt ID shared between the *repB* genes of pLMP1046 and pCTN1046 at 58.7%. The mosaic nature of the replication regions as well as the lack of nucleotide homology between the respective replication associated genes of the co-resident plasmids is a plausible explanation for the compatibility of the plasmids that co-exist in strain JCM1046. Several complete Toxin-Antitoxin (TA) systems were identified on plasmids pMP1046A and pLMP1046 and likely play a role in the stability and maintenance of the co-resident plasmids in JCM1046.

### Comparative *L. salivarius*genomics and relationship to phenotype

#### Chromosome

In contrast to the human probiotic strains *L. salivarius* UCC118 and *L. salivarius* CECT 5713 which share 98.5% nt pairwise ID between their chromosomes and 98.6% nt pairwise ID between their *repA*-type megaplasmids, the genome structure, and sequence of JCM1046 diverges significantly from the other published *L. salivarius* strains.

The chromosome of JCM1046 shares 68.4% nt pairwise ID with strain UCC118 and includes 55 regions (min 800 bp) [59], representing 16.5% of the chromosome, that are absent from strain UCC118 (Additional file 3). Indeed, a comparison of the chromosome of strain JCM1046 to that of the other published *L. salivarius* genome sequences revealed 48 chromosomally encoded genes in JCM1046 that were absent in the other published *L. salivarius* genomes (Additional file 4). These genes primarily belong to categories of genes that have been shown to be hypervariable among *L. salivarius* strains [7] and other *Lactobacillus* species [60] and include transposases, phage-associated genes, and genes involved in carbohydrate metabolism and host interaction (Additional file 4). The GC% map of the JCM1046 chromosome identifies three regions with significantly deviating GC content, one of which is the remnant prophage that is resident on the chromosome. The smallest of these regions stretches from residues 782,449 to 793,883 bp. This 11.4 kb region has a GC% content of 43.6% and encodes a protein containing a mucin-binding MucBP domain (LSJ_0784), several transposases, hypothetical proteins and a choloylglycine hydrolase (BSH2, LSJ_0788). Although present in the porcine strains JCM1046 and cp400, this region is absent from other sequenced genomes of *L. salivarius* and may represent a niche specific adaptation.

BSH2 is one of two choloylglycine hydrolase genes encoded by the genome of JCM1046 [11]; the second (BSH1, LSJ_2111) is present on pMP1046A and is widespread among *L. salivarius* strains [11]. In contrast, BSH2 has only been identified in three isolates to date, JCM1046, LMG14476 and cp400, all of which are of animal origins. BSH2 confers JCM1046 with an ability to resist much higher concentrations of the major human conjugated bile acids when compared to strains that harbour BSH1 alone [11]. In addition, BSH2 has recently been shown to reduce weight gain and serum LDL cholesterol and liver triglycerides in mice fed normal or high-fat diets [61].

We have previously shown that exopolysaccharide (EPS) production levels and the presence of associated genes vary widely in *L. salivarius* [7]. JCM1046 harbours a single EPS gene cluster that spans 33 kb, containing 33 genes, including two pseudogenes (Additional file 5). The EPS locus exhibits an atypical GC content relative to the rest of the chromosome, 29.7% and 33.1% respectively.

#### pMP1046A

Nine substantial regions of sequence diversity, ranging in size from 3.8-22.6 kb were identified between pMP1046A and the sequences of the other published *repA*-type megaplasmids (Figure 3; Table 2). Hypothetical proteins and transposases are abundant within these regions (Table 2). Indeed, region two and region four primarily harbour hypothetical proteins, while region six harbours only IS elements (Table 2, R2, R4 and R6). Regions three and eight mostly encode solute transporters (Table 2 R3 and R8).

**Table 2:** **Regions of sequence diversity in pMP1046A**

Region of diversity: base coordinates (***size bp***)	Gene	Start position	End position	Gene product
**R1: 14543..37152 (** ***22609*** **)**	LSJ_2012	14616	15683	Hypothetical membrane protein
	LSJ_2013	15827	16882	Hypothetical membrane protein
	LSJ_2014	17072	17242	Conserved hypothetical protein
	LSJ_2015	17257	18330	Putative thiosulfate sulfurtransferase
	LSJ_2016	18782	20686	D-proline reductase, *prdA*
	LSJ_2017	20688	21005	Conserved hypothetical protein
	LSJ_2018 (P)	20995	21720	Proline reductase, probable pseudogene
	LSJ_2019	21740	22480	Conserved hypothetical protein
	LSJ_2020	22501	22974	D-proline reductase
	LSJ_2021	22999	24018	Proline racemase
	LSJ_2022	24031	24909	Hypothetical membrane protein
	LSJ_2023	25005	26603	Amino acid permease
	LSJ_2024	26684	27322	Conserved hypothetical protein
	LSJ_2025	27434	27691	Conserved hypothetical protein
	LSJ_2026	27691	29583	Selenocysteine-specific elongation factor
	LSJ_2027	29573	30712	Cysteine desulfurase
	LSJ_2028	30713	32113	L-seryl-tRNA selenium transferase, *selA*
	LSJ_2029	32119	32448	Conserved hypothetical protein
	LSJ_2030	32560	33870	NADH dehydrogenase
	LSJ_2031	33963	34937	Selenophosphate synthase, *selD*
	LSJ_2032	35037	36209	Hypothetical membrane protein
	LSJ_2033	36228	36473	Conserved hypothetical protein
	LSJ_2034	36698	37129	Conserved hypothetical protein
**R2: 52540..64667 (** ***12127*** **)**	LSJ_2049	52523	54520	Conserved hypothetical protein
	LSJ_2050	54507	55370	Conserved hypothetical protein
	LSJ_2051	55360	56145	Conserved hypothetical protein
	LSJ_2052	56204	58906	DNA methylase
	LSJ_2053	58913	60871	DEAD/DEAH box helicase family protein
	LSJ_2054	60864	62066	Conserved hypothetical protein
	LSJ_2055	62137	64662	Conserved hypothetical protein
**R3: 88322..98017 (** ***9695*** **)**	LSJ_2078 (P)	88463	88739	Transposase, probable pseudogene
	LSJ_2079	88814	89641	Transposase ISLasa15, IS3 family
	LSJ_2080	90080	91279	MFS Transport protein
	LSJ_2081	91593	92813	MFS Transport protein
	LSJ_2082	92865	93761	Transcriptional regulators, LysR family
	LSJ_2083	93920	94756	Conserved hypothetical protein
	LSJ_2084	94785	95612	2-deoxy-D-gluconate 3-dehydrogenase
	LSJ_2085	95631	97346	Fumarate reductase flavoprotein subunit
	LSJ_2086	97367	98242	Shikimate 5-dehydrogenase
**R4: 100291..121050 (** ***20759*** **)**	LSJ_2089	100815	101621	Conserved hypothetical protein
	LSJ_2090	101614	101832	Hypothetical protein
	LSJ_2091	102071	102190	Hypothetical protein
	LSJ_2092	102310	103761	Plasmid replication protein-primase
	LSJ_2093	103865	104446	Hypothetical membrane protein
	LSJ_2094	104468	104620	Hypothetical membrane protein
	LSJ_2095	104818	105573	Hypothetical protein
	LSJ_2096	105746	106198	Hypothetical secreted protein
	LSJ_2097	106590	106853	Hypothetical protein
	LSJ_2098	106973	107965	Conserved hypothetical protein
	LSJ_2099	108439	108663	Hypothetical secreted protein, possible signal peptide
	LSJ_2100	109441	110277	Hypothetical protein
	LSJ_2101	110287	111057	Putative DNA-entry nuclease
	LSJ_2102	111064	111543	Conserved hypothetical protein
	LSJ_2103	111576	111743	Hypothetical secreted protein
	LSJ_2104	111993	112103	Hypothetical protein
	LSJ_2105	112160	112756	Conserved hypothetical protein
	LSJ_2106	112749	115094	Conserved hypothetical protein
	LSJ_2107	115914	118892	Hypothetical protein
	LSJ_2108	119031	120428	Conserved hypothetical protein
	LSJ_2109	120839	121078	Hypothetical protein
**R5: 147401..153337** ***(5936)***	LSJ_2136c	147401	148081	Fructose-6-phosphate aldolase
	LSJ_2137c	148148	148528	PTS system, glucitol/sorbitol-specific IIA component
	LSJ_2138c	148565	149590	PTS system, glucitol/sorbitol-specific IIBC component
	LSJ_2139c	149607	150149	PTS system, glucitol/sorbitol-specific IIC2 component
	LSJ_2140c	150161	150658	Sorbitol operon activator
	LSJ_2141c	150659	152518	Sorbitol operon transcription regulator
	LSJ_2142c	152534	153337	Sorbitol-6-phosphate 2-dehydrogenase
**R6: 160003..164289 (** ***4286*** **)**	LSJ_2150	160058	160575	Transposase ISLasa1a, IS1223 family
	LSJ_2151	160607	161473	IS1223 family transposase
	LSJ_2152	161537	162544	Transposase fragment
	LSJ_2153	162694	163983	ISL3 family transposase
**R7: 167503..182637 (** ***15134*** **)**	LSJ_2155	166716	167537	Integrase
	LSJ_2156	167573	167839	Hypothetical protein
	LSJ_2157	168343	169011	Hypothetical protein
	LSJ_2158	169087	169419	Hypothetical protein
	LSJ_2159	169424	170053	Conserved hypothetical protein
	LSJ_2160	170398	170802	Toxin antitoxin system, toxin component
	LSJ_2161	170802	171023	Toxin antitoxin system, antitoxin component
	LSJ_2162	171466	172614	AbpD bacteriocin export accessory protein
	LSJ_2163 (P)	1172630	174788	AbpT bacteriocin export accessory protein, probable pseudogene due to frameshift
	LSJ_2164	175441	175680	Hypothetical membrane spanning protein
	LSJ_2165	175717	176511	AbpR response regulator
	LSJ_2166	176525	177817	AbpK sensory Transduction Histidine Kinase
	LSJ_2167	177819	177938	AbpIP induction peptide
	LSJ_2168 (P)	178086	178232	AbpIM bacteriocin immunity protein
	LSJ_2169	178371	178577	Abp118 bacteriocin beta peptide
	LSJ_2170	178595	178789	Abp118 bacteriocin alpha peptide
	LSJ_2171	178795	179052	Bacteriocin-like prepeptide
	LSJ_2172	179182	179355	Nonfunctional salvaricin B precursor
	LSJ_2173	179588	179851	Hypothetical membrane spanning protein
	LSJ_2174	179890	180219	Hypothetical protein
	LSJ_2175	180441	181415	Hypothetical membrane associated protein
	LSJ_2176 (P)	1181578	182227	HAD-superfamily hydrolase, probable pseudogene due to frameshift
	LSJ_2177	182338	182679	Hypothetical protein
**R8: 189782..193560 (** ***3778*** **)**	LSJ_2187	189909	191414	Sodium solute symporter
	LSJ_2188	191432	192556	Na(+)/H(+) antiporter
	LSJ_2189	192623	193354	Xylose isomerase domain protein
**R9: 204232..215364 (** ***11132*** **)**	LSJ_2201	204295	206286	Transketolase
	LSJ_2202	206304	206957	Transaldolase
	LSJ_2203	207466	208521	L-iditol 2-dehydrogenase
	LSJ_2204	208562	209611	Alcohol dehydrogenase
	LSJ_2205	209625	210896	Galacitol PTS, EIIC
	LSJ_2206	210923	211219	Galactitol PTS, EIIB
	LSJ_2207	211254	211706	Galacitol PTS, EIIA
	LSJ_2208	211888	212688	DeoR family transcriptional regulator
	LSJ_2209	212812	215178	Xylulose-5-phosphate/fructose-6-phosphate phosphoketolase

Genes associated with the regions of diversity (R1-R9) in pMP1046A, as illustrated in Figure 3. Genes present on the reverse strand are denoted by the suffix c following the locus tag (LSJ_XXX). Pseudogenes are denoted by (P). Numbers in italics represents the size of the region in bp.

The largest region of diversity among the strains examined is 22.6 kb (Figure 3, R1) and harbours several genes predicted to work synergistically with chromosomally encoded pathways to broaden the metabolic capabilities of strain JCM1046*.* Although present in strain cp400, this region is highly divergent in all other examined plasmids (Figure 3) and primarily encodes proteins involved in aa metabolism. JCM1046 is prototrophic for L-proline due to the presence of a chromosomally-encoded pathway. Three paralogous genes (LSJ_2016, LSJ_2020 and LSJ_2021) in this region are responsible for the interconversion of L-proline to D-proline. Also present in this region are two genes (LSJ_2031, *selD* and LSJ_2028, *selA*) which work in conjunction with the chromosomally encode gene (LSJ_0220, *serS*) to synthesise L-selenocysteine. These increased biosynthetic capabilities are likely to enhance the ability of JCM1046 to thrive in the competitive porcine GIT.

The genes present in regions five and nine (Table 2) are primarily involved in the metabolism and transport of carbohydrates, and vary from strain to strain (Figure 3, R5 and R9). Similarly to pMP118, pMP1046A harbours both single copy and paralogous genes that complete a number of the carbohydrate fermentative pathways that are partially encoded by the chromosome of JCM1046A. These include the pentose phosphate and gluconeogenesis pathways as well as the fermentation pathways for sorbitol and rhamnose.

Bacteriocin production is a putative probiotic trait of *L. salivarius* strains (see review [62]). The genetic organisation of the 7.9 kb bacteriocin locus in pMP1046A is analogous to that of the Abp118 locus in the human isolate UCC118 (Figure 3 R7). The structural genes (LSJ_2170 and LSJ_2169) of the bacteriocin locus of pMP1046A, are identical to the genes (Sln1 and Sln2) which are responsible for the production of the two-component antilisterial bacteriocin Salivaricin P. This bacteriocin differs in sequence to Abp118 by two amino acids [63] and is produced by several other porcine isolates of *L. salivarius* [63, 64]. However, a frame-shift in the *abpT* gene (LSJ_2163) of JCM1046 is likely responsible for the bacteriocin negative phenotype observed in this strain [12].

#### pCTN1046

**Figure 4:**
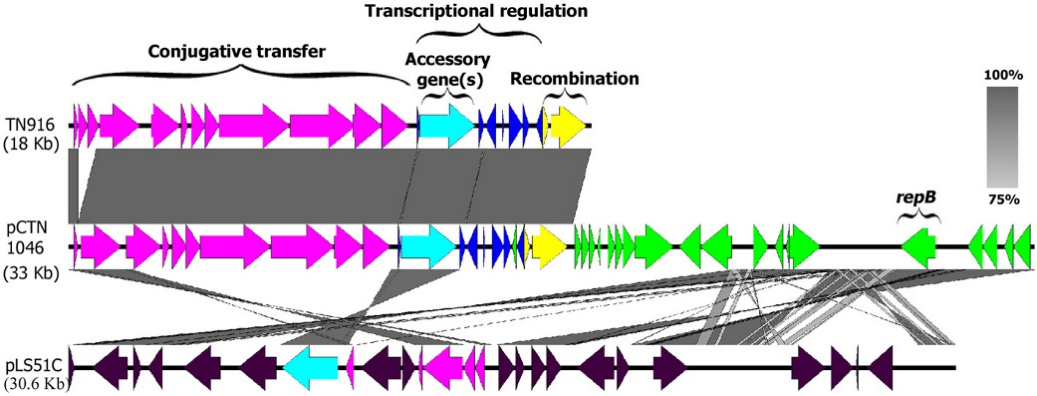
**Sequence alignment of Tn916, pCTN1046 and pLS51C.** A linear comparison of the BLASTN matches between the extrachromosomal replicons pCTN1046 and pLS51C (harboured by *L. salivarius* strain SMXD51 [16]) and the conjugative transposon Tn916. Vertical grey-coloured blocks between sequences indicate regions of shared nt ID. The gradient of the grey colour corresponds to the percentage of shared nt ID (dark grey (100%)-light grey (75%)). The genes in each element are coloured according to their function in the conjugative transposon Tn916: pink (conjugative transfer), turquoise (accessory genes and transcriptional regulation), dark blue (transcriptional regulation) and yellow (recombination). Genes encoded by the plasmid backbone of pCTN1046 are green, and those associated with the backbone of pLS51C are dark purple.

The conjugative element Tn6224 harboured by plasmid pCTN1046 shares 96.2% nt sequence ID with the conjugative element Tn916 and lacks only two genes which encode hypothetical proteins in the conjugative region of Tn916. When comparing pCTN1046 to other sequenced *L. salivarius* genomes, pCTN1046 shares 64.6% nt ID with the 30.4 kb plasmid pLS51C harboured by the probiotic avian isolate SMDX51 [16]. This plasmid shares sequence homology with both the plasmid backbone and conjugative element of pCTN1046 (Figure 4). Tn6224 appears to be functionally intact, containing the: conjugative, recombination, transcriptional regulation and accessory genes (Additional file 6) associated with Tn916. In contrast the integrated conjugative element that is resident in pSL51C appears to be a remnant of a conjugative element as it lacks the recombination genes *xis* (LSJ_5019) and *int* (LSJ_5020). pLS51C harbours a limited number of the conjugative genes present in Tn6224 and Tn916 but lacks the *ardA* gene present in pCTN1046 which has been recently shown to aid the transfer of mobile genetic elements (MGEs) between unrelated bacterial species [65]. A putative TnGBS1-like element (TnLsal1.1) was identified in *L. salivarius* strain DSM20555. However, our analysis suggests that the contig predicted to harbour TnLsal1.1 [66] forms part of the putative pMP20555 megaplasmid in the type-strain *L. salivarius* DSM20555. The weak homology between the proteins identified in TnLsal1.1 and those identified in other TnGBS1-like elements [66] may be due to their similar functional roles in their respective replicons.

*L. salivarius* strains are increasingly being examined for their probiotic properties in both humans and animals [5]. Dissemination of antibiotic resistance genes via the food chain to either the resident microbiota of the human gut or pathogenic bacteria is likely to have far reaching effects on both human and animal health and present a major financial cost [67]. Thus, the identification of conjugative transposons carrying antibiotic resistance genes in the genomes of two animal isolates of *L. salivarius* may have repercussions for strain selection in future probiotic studies.

#### pMP1046B and pLMP1046

Plasmids pMP1046B and pLMP1046 share neither sequence homology nor gene synteny with the additional *L. salivarius* plasmids sequenced to date. Both of these replicons require further functional characterisation to determine whether or not they have an impact on the phenotype and ecological properties of JCM1046.

## Conclusion

The porcine strain JCM1046 harbours the most structurally complex multipartite genome identified in *L. salivarius* to date. Through complete sequencing and assembly of the genome of JCM1046 we identified two additional replicons that were not previously known to form part of the plasmid complement of this strain, and that would probably not have been identified by the high-coverage draft genome sequencing commonly applied. We determined that one of these replicons, pMP1046B is a candidate chromid, though much of its gene function remains cryptic. The plasmids of *L. salivarius* probably confer on their host many of the genes associated with niche adaptation and which are known to modulate the phenotype of a strain significantly. JCM1046 was found to harbour both plasmid-encoded (pMP1046A) and chromosomally encoded genes associated with adaptation to the GIT environment. The putative replication *ori* of pLMP1046 was identified and the sequence of this linear plasmid will provide a genetic platform for the study of linear DNA replication in *Lactobacillus* sp. An integrated conjugative transposon (Tn6224), carrying tetracycline resistance was identified in plasmid pCTN1046, the first described in a sequenced *L. salivarius* genome. It will be interesting to see how prevalent Tn6224-like elements are within the *L. salivarius* population, as more genome sequences become available.

## Methods

### Bacterial strains and culture conditions

*L. salivarius* strains were routinely cultured at 37°C under micro-aerophilic conditions (5% CO_2_) in de Man-Rogosa-Sharpe (MRS) medium (Oxoid Ltd, Basingstoke, Hampshire, UK).

### PFGE plug preparations

Agarose gel plugs of high molecular weight DNA for PFGE were prepared according to a published protocol [12].

### S1-nuclease treatment

Single slices (2 mm × 2 mm) were treated with *Aspergillus oryzae* S1 nuclease (Roche, Mannheim, Germany) according to a published protocol [12].

### Restriction of PFGE plugs

Single slices (2 mm × 2 mm) were washed three times for 15 min in 1 ml 10 mM Tris.Cl, 0.1 mM EDTA (pH 8.0) at room temperature. Each slice was pre-incubated with 250 μl of restriction buffer recommended for the enzyme for 30 min at 4°C and then replaced with 250 μl of fresh buffer containing 20 units of restriction enzyme. Restriction digests were carried out overnight at temperatures recommended by the supplier.

### Pulsed field gel electrophoresis

Treated (S1-nuclease/restriction enzyme) and untreated plugs of genomic DNA were examined under conditions employed in a previously published protocol [12]. Gels were stained in distilled water containing 0.5 μg/ml ethidium bromide for 60 min in light-limited conditions and destained in water for 30 min.

### Probe preparation and Southern hybridization

Probe preparations and Southern blot hybridizations were carried out according to a published protocol [12]. The primers used to generate PCR amplicons that were used as probes are listed in Additional file 7.

### Genome sequencing

*L. salivarius* genomic DNA (gDNA) isolation was performed as described previously [1]. The genome of JCM1046 genome was sequenced using a combination of shotgun sequencing by the Sanger method (4-fold coverage), pyrosequencing (24-fold coverage) and Illumina (204-fold coverage). A large-insert (~40 kb) fosmid library was constructed in the CopyControl™ pCCFOS™ vector system (Epicentre Technologies, USA).Corporation, USA) Insert ends (~800 bp/read) were sequenced generating mate pairs and 7.5 Mb sequencing data. Pyrosequencing generated approximately 217,000 unpaired reads (~250 nt); from a half plate on a 454 FLX instrument (Agencourt Biosciences, Beverly, MA). In addition to the shotgun and 454 data for the JCM1046 genome, an additional half lane of Illumina sequencing (23 Mb total sequence data) was obtained which consisted of a 3 kb mate-pair library and a 400 bp paired-end library (Fasteris, Geneva, Switzerland). Each Illumina library provided an average of 204-fold coverage. Illumina reads were assembled (default settings) into contigs using Velvet v 0.7 [68], which were then used to generate 300 bp pseudocontigs. A *de novo* genome assembly of the shotgun, 454 and Illumina (pseudocontigs) sequence data was performed using the Roche/454 Life Sciences Newbler (Gs) assembler v 2.3 [69], producing an initial assembly of 102 contigs (>500 bp) distributed over 32 scaffolds for the genome of JCM1046. The resulting 454 assembly was then used as a reference for the mapping of raw Illumina data. This mapping assembly was performed using Mira [70] and undertaken to extend contigs, close gaps and for error correction of the draft genome. Gap closure was achieved using a PCR-based strategy. Primers were designed at the end of contigs and Dreamtaq DNA polymerase (Fermentas, Ontario, Canada) was used to amplify products corresponding to contig-contig gaps. Scaffolds were ordered and oriented by PCR using primers were designed at the ends of the scaffolds and the inter-scaffold region was amplified using Extensor long PCR enzyme mix (Abgene, Epsom, UK). PCR products for both the sequencing gaps and the inter-scaffold gaps were sequenced by Eurofins MWG Operon (Ebersberg, Germany) and the sequences were integrated into the assembly using PHRAP [71]. Correct placement of the gap sequences was confirmed by observation using Tablet, a next generation sequencing graphical viewer [72].

### Genome annotation

Annotation was carried out according to a published protocol [73] with minor modifications. Specifically, initial annotation was transferred from the related strain *L. salivarius* UCC118 [74] and then manually curated in Artemis [75] *.* PHAST [48] was used to identify prophage regions within the genome sequence.

### Data availability

The annotated genome sequence has been deposited in GenBank under accession numbers CP007646 (chromosome), CP007647 (pMP1046A), CP007648 (pMP1046B), CP007649 (pLMP1046), CP007650 (pCTN1046).

### Genome comparisons

Nucleotide alignments were generated using a local BLAST v 2.2.22 installation which were then visualized and analyzed for gene conservation and sequence synteny using the Artemis Comparison Tool (ACT) [76].

### Identification of novel genetic regions

The Novel Region Finder module of Pan seq v 2.0 [59] was used to identify novel genomic regions in strain JCM1046, compared to other *L. salivarius* genome sequences. A minimum novel region size of 800 bp was chosen and default Nucmer values were used.

## Electronic supplementary material

Additional file 1: **Pseudogenes in the** ***L. salivarius*** **JCM1046 genome.** Pseudogenes were characterized as such due to the presence of in-sequence frame-shifts, deletions, or interruptions of the gene by insertion sequences (IS). Genes present on the reverse strand are denoted by the suffix c following the locus tag (LSJ_XXX). (XLSX 14 KB)

Additional file 2: **Transposable elements and insertion sequence (IS) elements in the** ***L. salivarius*** **JCM1046 genome.** Pseudogenes are denoted by (P). Genes present on the reverse strand are denoted by the suffix c following the locus tag (LSJ_XXX). (XLSX 12 KB)

Additional file 3: **Genes harboured by the chromosome of strain JCM1046 that are absent from the chromosome of UCC118.** Regions of diversity were determined using Panseq [59]. Pseudogenes are denoted by (P). Genes present on the reverse strand are denoted by the suffix c following the locus tag (LSJ_XXX). (XLSX 18 KB)

Additional file 4: **Genes harboured by the chromosome of strain JCM1046 that are absent from other** ***L. salivarius*** **sequenced genomes.** Gene presence/absence was determined by BLASTP sequence comparisons. Pseudogenes are denoted by (P). Genes present on the reverse strand are denoted by the suffix c following the locus tag (LSJ_XXX). (XLSX 10 KB)

Additional file 5: **Exopolysaccharide gene cluster present on the chromosome of** ***Lactobacillus salivarius*** **JCM1046.** Pseudogenes are denoted by (P). Genes present on the reverse strand are denoted by the suffix c following the locus tag (LSJ_XXX). (XLSX 11 KB)

Additional file 6: **Genes harboured by pCTN1046.** Pseudogenes are denoted by (P). Genes present on the reverse strand are denoted by the suffix c following the locus tag (LSJ_XXX). (XLSX 11 KB)

Additional file 7: **Primers used to generate Southern Hybridization probes.** (XLSX 9 KB)

## Abbreviations

aa: Amino acid
ACT: Artemis comparison tool
BLAST: Basic local alignment search tool
bp: Base pairs
CRISPR: Clustered regularly interspaced short palindromic repeats
CAS: CRISPR-associated sequence
DR: Direct repeat
EPS: Exopolysaccharide
GIT: Gastrointestinal tract
ID: Identity
IS: Insertion sequence
LAB: Lactic acid bacteria
LDL: Low-density lipoprotein
NCBI: National center for biotechnology information
PCR: Polymerase chain reaction
nr: Nonredundant protein database
nt: Nucleotides
sp: Species
TIR: Terminal inverted repeat.

## References

[1] Li Y, Raftis E, Canchaya C, Fitzgerald GF, Sinderen DV, O’Toole PW (2006). Polyphasic analysis indicates that *Lactobacillus salivarius* subsp. *salivarius* and *Lactobacillus salivarius* subsp. *salicinius* do not merit separate subspecies status. Int J of Syst Evol Microbiol.

[2] Reuter G (2001). The *Lactobacillus* and *Bifidobacterium* microflora of the human intestine: composition and succession. Curr Issues Intest Microbiol.

[3] Mitsuoka T (1969). Vergleichende untersuchungen Über die *Laktobazillen* aus den faeces von menschen, schweinen und hÜhnern. Bakteriol.

[4] Martín R, Jiménez E, Olivares M, Marín ML, Fernández L, Xaus J, Rodríguez JM (2006). *Lactobacillus salivarius* CECT 5713, a potential probiotic strain isolated from infant feces and breast milk of a mother-child pair. Int J Food Microbiol.

[5] Neville BA, O'Toole PW (2010). Probiotic properties of *Lactobacillus salivarius* and closely related *Lactobacillus* species. Future Microbiol.

[6] Ryan KA, Daly P, Li Y, Hooton C, O'Toole PW (2008). Strain-specific inhibition of *Helicobacter pylori* by *Lactobacillus salivarius* and other lactobacilli. J Antimicrob Chemother.

[7] Raftis EJ, Salvetti E, Torriani S, Felis GE, O'Toole PW (2011). Genomic diversity of *Lactobacillus salivarius*. Appl Environ Microbiol.

[8] Flynn S, van Sinderen D, Thornton GM, Holo H, Nes IF, Collins JK (2002). Characterization of the genetic locus responsible for the production of ABP-118, a novel bacteriocin produced by the probiotic bacterium *Lactobacillus salivarius* subsp. *salivarius* UCC118. Microbiology.

[9] Claesson MJ, Li Y, Leahy S, Canchaya C, van Pijkeren JP, Cerdeño-Tárraga AM, Parkhill J, Flynn S, O’Sullivan GC, Collins JK, Higgins D, Shanahan F, Fitzgerald GF, van Sinderen D, O'Toole PW (2006). Multireplicon genome architecture of *Lactobacillus salivarius*. Proc Natl Acad Sci.

[10] Corr SC, Li Y, Riedel CU, O'Toole PW, Hill C, Gahan CGM (2007). Bacteriocin production as a mechanism for the antiinfective activity of *Lactobacillus salivarius* UCC118. Proc Natl Acad Sci.

[11] Fang F, Li Y, Bumann M, Raftis EJ, Casey PG, Cooney JC, Walsh MA, O'Toole PW (2009). Allelic variation of bile salt hydrolase genes in *Lactobacillus salivarius* does not determine bile resistance levels. J Bact.

[12] Li Y, Canchaya C, Fang F, Raftis E, Ryan KA, van Pijkeren J-P, van Sinderen D, O'Toole PW (2007). Distribution of megaplasmids in *Lactobacillus salivarius* and other lactobacilli. J Bacteriol.

[13] van Pijkeren J, Canchaya C, Ryan K, Li Y, Claesson M, Sheil B, Steidler L, O'Mahony L, Fitzgerald G, van Sinderen D (2006). Comparative and functional analysis of sortase-dependent proteins in the predicted secretome of *Lactobacillus salivarius* UCC118. Appl Environ Microbiol.

[14] Jimenez E, Martin R, Maldonado A, Martin V, Gomez de Segura A, Fernandez L, Rodriguez JM (2010). Complete genome sequence of *Lactobacillus salivarius* CECT 5713, a probiotic strain isolated from human milk and infant feces. J Bacteriol.

[15] Ham J-S, Kim H-W, Seol K-H, Jang A, Jeong S-G, Oh M-H, Kim D-H, Kang D-K, Kim G-B, Cha C-J (2011). Genome sequence of *Lactobacillus salivarius* NIAS840, isolated from chicken intestine. J Bacteriol.

[16] Kergourlay G, Messaoudi S, Dousset X, Prévost H (2012). Genome Sequence of *Lactobacillus salivarius* SMXD51, a Potential Probiotic Strain Isolated from Chicken Cecum, Showing Anti-*Campylobacter* Activity. J Bacteriol.

[17] Cho Y-J, Choi JK, Kim J-H, Lim Y-S, Ham J-S, Kang D-K, Chun J, Paik H-D, Kim G-B (2011). Genome sequence of *Lactobacillus salivarius* GJ-24, a probiotic strain isolated from healthy adult intestine. J Bacteriol.

[18] MacKenzie DA, McLay K, Roos S, Walter J, Swarbreck D, Drou N, Crossman LC, Juge N (2014). Draft Genome Sequence of a Novel *Lactobacillus salivarius* Strain Isolated from Piglet. Genome Announcements.

[19] Overhage J, Sielker S, Homburg S, Parschat K, Fetzner S (2005). Identification of large linear plasmids in Arthrobacter spp. encoding the degradation of quinaldine to anthranilate. Microbiology.

[20] Kinashi H (2011). Giant linear plasmids in *Streptomyces*: a treasure trove of antibiotic biosynthetic clusters. J Antibiot.

[21] Chater K, Kinashi H, Meinhardt F, Klassen R (2007). *Streptomyces*; linear plasmids: their discovery, functions, interactions with other replicons, and evolutionary significance. Microbial Linear Plasmids, Volume 7.

[22] Chen C, Meinhardt F, Klassen R (2007). *Streptomyces*; linear plasmids: replication and telomeres. Microbial Linear Plasmids, Volume 7.

[23] Barbour A, Garon C (1987). Linear plasmids of the bacterium *Borrelia burgdorferi* have covalently closed ends. Science.

[24] Stromsten NJ, Benson SD, Burnett RM, Bamford DH, Bamford JKH (2003). The *Bacillus thuringiensis* linear double-stranded DNA phage Bam35, which Is highly similar to the *Bacillus cereus* linear plasmid pBClin15, has a prophage state. J Bacteriol.

[25] Ravin NV (2011). N15: The linear phage-plasmid. Plasmid.

[26] Hertwig S, Klein I, Lurz R, Lanka E, Appel B (2003). PY54, a linear plasmid prophage of *Yersinia enterocolitica* with covalently closed ends. Mol Microbiol.

[27] Casjens SR, Gilcrease EB, Huang WM, Bunny KL, Pedulla ML, Ford ME, Houtz JM, Hatfull GF, Hendrix RW (2004). The pKO2 linear plasmid prophage of *Klebsiella oxytoca*. J Bacteriol.

[28] Alemayehu D, Ross RP, O'Sullivan O, Coffey A, Stanton C, Fitzgerald GF, McAuliffe O (2009). Genome of a virulent bacteriophage Lb338-1 that lyses the probiotic *Lactobacillus paracasei* cheese strain. Gene.

[29] Roussel Y, Colmin C, Simonet JM, Decaris B (1993). Strain characterization, genome size and plasmid content in the *Lactobacillus acidophilus* group (Hansen and Mocquot). J Appl Bacteriol.

[30] Franke AE, Clewell DB (1981). Evidence for a chromosome-borne resistance transposon (Tn916) in *Streptococcus faecalis* that is capable of "conjugal" transfer in the absence of a conjugative plasmid. J Bacteriol.

[31] Clewell DB, Flannagan SE, Jaworski DD (1995). Unconstrained bacterial promiscuity: the Tn916-Tn1545 family of conjugative transposons. Trends Microbiol.

[32] Bertram J, Stratz M, Durre P (1991). Natural transfer of conjugative transposon Tn916 between gram-positive and gram-negative bacteria. J Bacteriol.

[33] Boguslawska J, Zycka-Krzesinska J, Wilcks A, Bardowski J (2009). Intra- and interspecies conjugal transfer of Tn916-like elements from *Lactococcus lactis in vitro* and *in vivo*. Appl Environ Microbiol.

[34] Devirgiliis C, Coppola D, Barile S, Colonna B, Perozzi G (2009). Characterization of the Tn916 conjugative transposon in a food-borne strain of *Lactobacillus paracasei*. Appl Environ Microbiol.

[35] Roberts AP, Cheah G, Ready D, Pratten J, Wilson M, Mullany P (2001). Transfer of Tn916-like elements in microcosm dental plaques. Antimicrob Agents Chemother.

[36] Schjørring S, Krogfelt KA (2010). Assessment of bacterial antibiotic resistance transfer in the gut. Int J Microbiol.

[37] Wozniak R, Waldor M (2010). Integrative and conjugative elements: mosaic mobile genetic elements enabling dynamic lateral gene flow. Nature reviews Microbiology.

[38] Roberts AP, Mullany P (2009). A modular master on the move: the Tn916 family of mobile genetic elements. Trends Microbiol.

[39] Carver T, Thomson N, Bleasby A, Berriman M, Parkhill J (2009). DNAPlotter: circular and linear interactive genome visualization. Bioinformatics.

[40] Fang F, Flynn S, Li Y, Claesson MJ, van Pijkeren J-P, Collins JK, van Sinderen D, O'Toole PW (2008). Characterization of endogenous plasmids from *Lactobacillus salivarius* UCC118. Appl Environ Microbiol.

[41] Barton BM, Harding GP, Zuccarelli AJ (1995). A general method for detecting and sizing large plasmids. Analyst Biochem.

[42] Warner JE, Onderdonk AB (2003). Method for Optimizing Pulsed-Field Gel Electrophoresis Banding Pattern Data. JMD.

[43] Wang H, Roberts AP, Mullany P (2000). DNA sequence of the insertional hot spot of Tn916 in the *Clostridium difficile* genome and discovery of a Tn916-like element in an environmental isolate integrated in the same hot spot. FEMS Microbiol Lett.

[44] Harrison PW, Lower RPJ, Kim NKD, Young JPW (2010). Introducing the bacterial chromid”: not a chromosome, not a plasmid. Trends Microbiol.

[45] Sanchez-Perez G, Mira A, Nyirő G, Pašić L, Rodriguez-Valera F (2008). Adapting to environmental changes using specialized paralogs. Trends Genet.

[46] Wagenknecht M, Dib J, Thürmer A, Daniel R, Farías M, Meinhardt F (2010). Structural peculiarities of linear megaplasmid, pLMA1, from *Micrococcus luteus*; interfere with pyrosequencing reads assembly. Biotechnol Lett.

[47] Roberts AP, Chandler M, Courvalin P, Guédon G, Mullany P, Pembroke T, Rood JI, Jeffery Smith C, Summers AO, Tsuda M, Berg DE (2008). Revised nomenclature for transposable genetic elements. Plasmid.

[48] Zhou Y, Liang Y, Lynch KH, Dennis JJ, Wishart DS (2011). PHAST: A fast phage search tool. Nucleic Acids Res.

[49] Jore MM, Brouns SJJ, van der Oost J: **RNA in Defense: CRISPRs Protect Prokaryotes against Mobile Genetic Elements.** *Cold Spring Harb Perspect Biol* 2012.,**4**(6)**:** http://cshperspectives.cshlp.org/content/4/6/a003657.full.pdf+html 10.1101/cshperspect.a003657 PMC3367551 21441598

[50] Jannière L, Gruss A, Ehrlich D, Sonenshein JAH AL, Losick R (1993). Plasmids. Bacillus subtilis and other gram-positive bacteria.

[51] Alikhan N-F, Petty N, Ben Zakour N, Beatson S (2011). BLAST Ring Image Generator (BRIG): simple prokaryote genome comparisons. BMC Genomics.

[52] Bentley SD, Parkhill J (2004). Comparative genomic structure of prokaryotes. Annu Rev Genet.

[53] Heavens D, Tailford LE, Crossman L, Jeffers F, MacKenzie DA, Caccamo M, Juge N (2011). Genome Sequence of the Vertebrate Gut Symbiont *Lactobacillus reuteri* ATCC 53608. J Bacteriol.

[54] Beaurepaire C, Chaconas G (2005). Mapping of essential replication functions of the linear plasmid lp17 of *B. burgdorferi* by targeted deletion walking. Mol Microbiol.

[55] Chang P-C, Cohen SN (1994). Birdirectional replication from an internal origin in a linear *Streptomyces* plasmid. Science.

[56] Ravin NV, Kuprianov VV, Gilcrease EB, Casjens SR (2003). Bidirectional replication from an internal ori site of the linear N15 plasmid prophage. Nucleic Acids Res.

[57] Mardanov A, Ravin N (2006). Functional characterization of the *repA* replication gene of linear plasmid prophage N15. Res Microbiol.

[58] Shiffman D, Cohen SN (1992). Reconstruction of *Streptomyces* linear plasmid replication from separately cloned DNA fragment: existence pf a cryptic origin of circular replication within the linear plasmid. Proc Natl Acad Sci U S A.

[59] Laing C, Buchanan C, Taboada E, Zhang Y, Kropinski A, Villegas A, Thomas J, Gannon V (2010). Pan-genome sequence analysis using Panseq: an online tool for the rapid analysis of core and accessory genomic regions. BMC Bioinformatics.

[60] Berger B, Pridmore R, Barretto C, Delmas-Julien F, Schreiber K, Arigoni F, Brüssow H (2007). Similarity and Differences in the *Lactobacillus acidophilus* Group Identified by Polyphasic Analysis and Comparative Genomics. J Bacteriol.

[61] Joyce SA, MacSharry J, Casey PG, Kinsella M, Murphy EF, Shanahan F, Hill C, Gahan CGM (2014). Regulation of host weight gain and lipid metabolism by bacterial bile acid modification in the gut. Proc Natl Acad Sci.

[62] Messaoudi S, Manai M, Kergourlay G, Prévost H, Connil N, Chobert JM, Dousset X (2013). *Lactobacillus salivarius*: Bacteriocin and probiotic activity. Food Microbiol.

[63] Barrett E, Hayes M, O'Connor P, Gardiner G, Fitzgerald GF, Stanton C, Ross RP, Hill C (2007). Salivaricin P, one of a family of two-component antilisterial bacteriocins produced by intestinal isolates of *Lactobacillus salivarius*. Appl Environ Microbiol.

[64] O'Shea EF, O'Connor PM, Raftis EJ, O'Toole PW, Stanton C, Cotter PD, Ross RP, Hill C (2011). Production of Multiple Bacteriocins from a Single Locus by Gastrointestinal Strains of *Lactobacillus salivarius*. J Bacteriol.

[65] Chen K, Reuter M, Sanghvi B, Roberts GA, Cooper LP, Tilling M, Blakely GW, Dryden DTF (2014). ArdA proteins from different mobile genetic elements can bind to the EcoKI Type I DNA methyltransferase of *E. coli* K12. Biochimica et Biophysica Acta (BBA) - Proteins and Proteomics.

[66] Guérillot R, Da Cunha V, Sauvage E, Bouchier C, Glaser P (2013). Modular evolution of TnGBSs, a new family of integrative and conjugative elements associating insertion sequence transposition, plasmid replication, and conjugation for their spreading. J Bacteriol.

[67] Panel F (2005). Opinion of the Scientific Panel on additives and products or substances used in animal feed (FEEDAP) on the updating of the criteria used in the assessment of bacteria for resistance to antibiotics of human or veterinary importance. EFSA J.

[68] Zerbino DR, Birney E (2008). Velvet: Algorithms for de novo short read assembly using de Bruijn graphs. Genome Res.

[69] Mardis ER (2008). Next-Generation DNA Sequencing Methods. Annu Rev Genomics Hum Genet.

[70] Chevreux B, Wetter TSS (1999). Genome sequence assembly using trace signals and additional sequence information. Computer Science and Biology: Proceedings of the German Conference on Bioinformatics (GCB).

[71] Green P (1999). PHRAP v 1.080812.

[72] Milne I, Bayer M, Cardle L, Shaw P, Stephen G, Wright F, Marshall D (2010). Tablet—next generation sequence assembly visualization. Bioinformatics.

[73] Forde B, Neville B, O' Donnell M, Riboulet-Bisson E, Claesson M, Coghlan A, Ross R, O' Toole P (2011). Genome sequences and comparative genomics of two *Lactobacillus ruminis* strains from the bovine and human intestinal tracts. Microb Cell Fact.

[74] Otto TD, Dillon GP, Degrave WS, Berriman M (2011). RATT: Rapid Annotation Transfer Tool. Nucleic Acids Res.

[75] Rutherford K, Parkhill J, Crook J, Horsnell T, Rice P, Rajandream M-A, Barrell B (2000). Artemis: sequence visualization and annotation. Bioinformatics.

[76] Carver TJ, Rutherford KM, Berriman M, Rajandream MA, Barrell BG, Parkhill J (2005). ACT: the Artemis comparison tool. Bioinformatics.

